# KIFC1: a promising chemotherapy target for cancer treatment?

**DOI:** 10.18632/oncotarget.8799

**Published:** 2016-04-19

**Authors:** Yu-Xi Xiao, Wan-Xi Yang

**Affiliations:** ^1^ The Sperm Laboratory, College of Life Sciences, Zhejiang University, Hangzhou, China

**Keywords:** kinesin, KIFC1, chemotherapy target, cancer

## Abstract

The kinesin motor KIFC1 has been suggested as a potential chemotherapy target due to its critical role in clustering of the multiple centrosomes found in cancer cells. In this regard, KIFC1 seems to be non-essential in normal somatic cells which usually possess only two centrosomes. Moreover, KIFC1 is also found to initiatively drive tumor malignancy and metastasis by stabilizing a certain degree of genetic instability, delaying cell cycle and protecting cancer cell surviving signals. However, that KIFC1 also plays roles in other specific cell types complicates the question of whether it is a promising chemotherapy target for cancer treatment. For example, KIFC1 is found functionally significant in vesicular and organelle trafficking, spermiogenesis, oocyte development, embryo gestation and double-strand DNA transportation. In this review we summarize a recent collection of information so as to provide a generalized picture of ideas and mechanisms against and in favor of KIFC1 as a chemotherapy target. And we also drew the conclusion that KIFC1 is a promising chemotherapy target for some types of cancers, because the side-effects of inhibiting KIFC1 mentioned in this review are theoretically easy to avoid, while KIFC1 is functionally indispensable during mitosis and malignancy of multi-centrosome cancer cells. Further investigations of how KIFC1 is regulated throughout the mitosis in cancer cells are needed for the understanding of the pathways where KIFC1 is involved and for further exploitation of indirect KIFC1 inhibitors.

## INTRODUCTION

Kinesin-14 family proteins are C-terminal kinesins with special minus end motility on microtubules. Thus far, only three members of this family, HsKIFC1 (HSET), HsKIFC2, and HsKFC3, have been discovered in humans [1–5]. Among those, most attention has been focusing on KIFC1 which is involved in spindle pole organization [6]. NCD in *Drosophil*a, XCTK2 in *Xenopus*, and Kar3 in *Saccharomyces* are known homologues for HsKIFC1 [4].

The organization of the mitotic spindle is a pivotal target for anti-cancer therapy due to the significantly higher duplication rates of cancer cells as compared to normal cells. [7, 8]. However, this method is limited. Determining the correct dosage is a key issue. On the one hand, overdose will lead to complications as the tubulins of normal cells will be adversely affected, on the other, with insufficient dose the motility and positioning of the microtubules is likely to be rescued by other factors, possibly leading to drug resistance and ineffective treatment. Therefore, an alternative strategy has emerged that directly targets kinesins on the microtubules. This new strategy has achieved some progress so far. Inhibitors targeting Eg5 from kinesin 5 [9–13] and CENP-E from the kinesin 7 family [14] have progressed into clinical trials and positive reports have been published. It seems likely that the next focus in this area will be upon HsKIFC1 (HSET) as it is considered to be redundant in normal somatic cells, yet indispensable for the proper division of cancer cells [15, 16]. However, the other roles that KIFC1 may play during vesicular and organelle trafficking [17], spermiogenesis [18, 19], oocyte development [20], and double-strand DNA transportation [21], continue to raise concerns about putting KIFC1 inhibitors into clinical usage.

## EVIDENCE FOR APPLICABILITY OF KIFC1-RELATED SYSTEMS IN ANTI-CANCER TREATMENT

### Elevated expression in a variety of cancer tissues

Despite other physiological functions and additional roles, both known and as yet unknown, that kinesin-14 has in reproductive systems and in various specific cell types, in humans their expression remains most notably visible as accomplishing assistance in the metastasis and survival of cancer cells. It is reported that KIFC1 is abundantly and widely expressed in cancer cells of the ovary [22], breast [23], bladder [24], lung [25], kidney [26] and other cancers [26]. The special status of KIFC1 in cancer cells is, therefore, well established. However, whilst many of the details of exactly how the elevated expression of KIFC1 occurs, remains unclear, part of the story, is beginning to be elucidated. The discovery, in breast cancer tissue, that the amplification of the KIFC1 gene relative to centromeres of chromosome 1 in breast cancer tissue gave the first hint as to why such a high expression of KIFC1 seems to be required in cancer cells [23].

How those amplified KIFC1 genes transcribe in cancer cells need further demonstration. But it seems likely in estrogen-receptor positive tumor cells with multi-centrosomes, that a probable transcription and up-regulation pathway of KIFC1 is as follows. Firstly, E2alpha, able to receive and be stimulated by E2 (estrogen-17beta-estradiol), triggers E2F to cooperate with p110 CUX1 and directly upregulate an aberrant expression of the kifc1 gene as a transcription factor [27, 28]. In addition, the stimulation of E2alpha is also involved in the activation of ANCCA (AAA nuclear coregulator cancer associated), a bromodomain containing ATPase protein, whose suppression remarkably diminishes the E2 induced effect of KIFC1. This indicates the ANCCA's possible up-regulation role in cases of KIFC1overexpression. Further experiments also demonstrate that the E2 induction of overexpression of other kinesins including KIF4A, KIF15, KIF20A, and KIF23, is marked by methylation under the mediation of ANCCA at histones near the gene promoters of those kinesins. The methylation is accomplished by corresponding histone methyltransferase MLL [29]. This indicates the possibility of the same methylation near the histones of the KIFC1 gene promoter to accomplish an increased KIFC1 expression.

### Tumor metastasis

Metastasis is one of the most concerning features of cancer. In non-small-cell lung cancer (NSCLC), KIFC1 is defined to be a critical positive indicator of brain metastasis by real-time quantitative reverse transcriptase PCR screening analysis, occurring second in line beyond that of CDH2 (N-Cadherin) [25]. Similarly, KIFC1 is also noted as a candidate for a metastases onset marker as indicated by ovarian cancers. This was shown by *in silico* gene expression database analysis [22]. Detailed mechanisms are still unknown but our suspicion is that KIFC1 may alter the cytoskeleton of the cancer cells by stabilizing the survival of multi-centrosome clustering cells so as to enhance cancer cell polarity, and hence powering the epithelial-mesenchymal-transition (EMT) and enhance cell motility [30].

### Centrosome clustering function and tumor malignancy

In most animal cells, the single pair of centrosomes are thought to be a location of the microtubule organization center (MTOC) which is formed at the poles of the bipolar spindle during mitosis [31]. The process whereby cells possess more than two centrosomes is referred to as centrosome amplification. This is designated as a primary characteristic of cancers and is correlated with increased tumor grade [30, 32, 33] and chromosomal instability [34–37]. Genetic instability levels rise along with the ponderance of centrosome amplification [38–39]. This, therefore, paves the way for tumorigenesis. The idea that ascending centrosome amplification levels lead to tumor genesis has been concluded from studies in flies [40]. In addition, murine studies show that adverse genetic conditions of cells, such as in the cases of aneuploidy, can also raise the likelihood of cancer formation in mammals [41]. Centrosome amplification, therefore, has the capacity to cause genetic instability and is a hallmark of most cancer cells.

**Figure 1:**
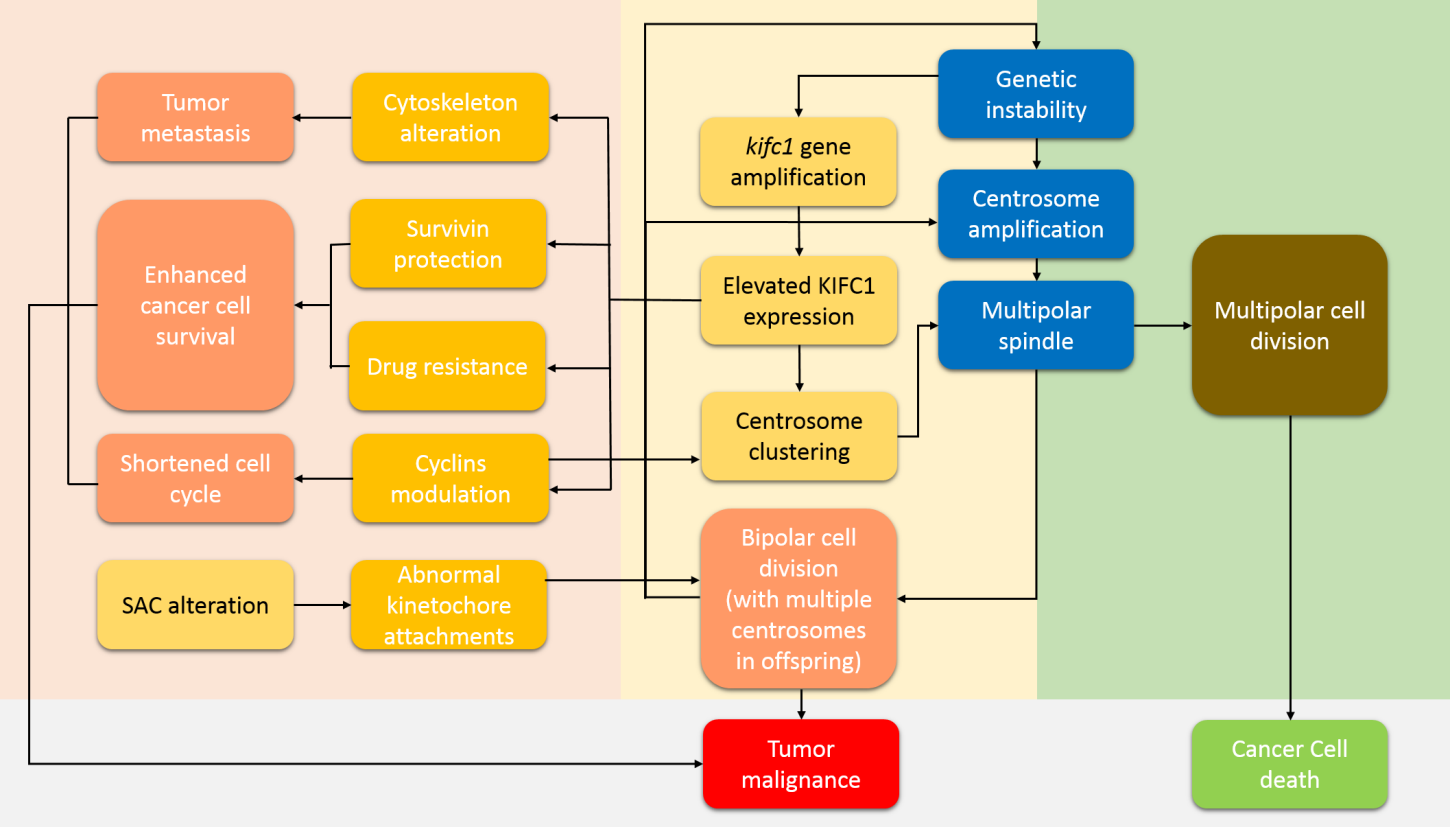
How KIFC1 drives tumor malignancy and prevents cancer cell death This pathway can be roughly divided into four areas. The grey area shows the final cancer cell condition. The light orange area shows a positive feedback cycle which we name the KIFC1-driven tumor malignancy cycle. Genetic instability induces centrosome amplification, which is a feature of cancer cells and enhances tumor malignancy, as well as kifc1 gene amplification [23]. Centrosome amplification induces the formation of multipolar spindles. Any failure in the turning of multipolar spindles into bipolar ones will eventually lead to cell death (the light green area) [42]. However, increased KIFC1 expression rescues cancer cells from such a death by assisting cells with multipolar spindle stability and by helping to achieve proper bipolar segregation through centrosome clustering activity [16, 40]. A proper bipolar cell division further stabilizes any genetic instability. Hence a positive feedback cycle is completed. The pink area illustrates other pathways of how KIFC1 drive tumor metastasis, enhance cell survival, and shorten the cell cycle so as to speed up cell proliferation.

Nevertheless, the existence of multiple centrosomes can be lethal. Missegregation and aneuploidy might occur along with the formation of the multipolar spindle [42]. How cancer cells rescue themselves from this paradox still needs to be understood. Unsurprisingly, cancer cells with supernumerary centrosomes still find their way to proliferate [43, 44]. There are several hypotheses on how they solve this problem, one of which describes that multiple MTOCs may be clustering together at the prometaphase. This hypothesis is based on the fact that in genetically unstable cancer cells, single kinetochores are capable of attaching to microtubules coming from different MTOCs [45]. The clustered MTOCs function is to forms two supernumerary centrosomes and a single pseudo-bipolar spindle at the metaphase [43, 44, 46]. This has been observed in altered mouse neuroblastoma cell lines [44] and in all kinds of tumors [38]. Via this kind of centrosome clustering mechanism, KIFC1, which is able to be activated by genetic instability signals as in DNA-damaging [47], rescues cancer cells from aneuploidy and cell death [16, 40]. Thus, the lowered missegregation level ensures the survival of cancer cells with a certain extent of genetic instability, which in turn increases the chance of mutations and enhances tumor malignancy. However, how those cells with DNA-damage escape from the G2 phase arrest, remains unclear. That lowered missegregation levels of cancer cells actually increases overall genetic instability of the cancer cell group has been verified in ovarian cancer [22], and breast cancer [23]. It has been reasoned that KIFC1 might actually drive malignancy in cancers instead of just acting as a “beacon” for it [48]. This idea has already been proven in breast cancer cells where KIFC1 not only enhances cell survival but also increases chances of genetic mutation by assisting the proper cell division of multi-centrosome cancer cells *via* its centrosome clustering activity [23]. A positive feedback to KIFC1-driven tumor malignancy by KIFC1's centrosome clustering activity is proposed in Figure 1.

**Figure 2:**
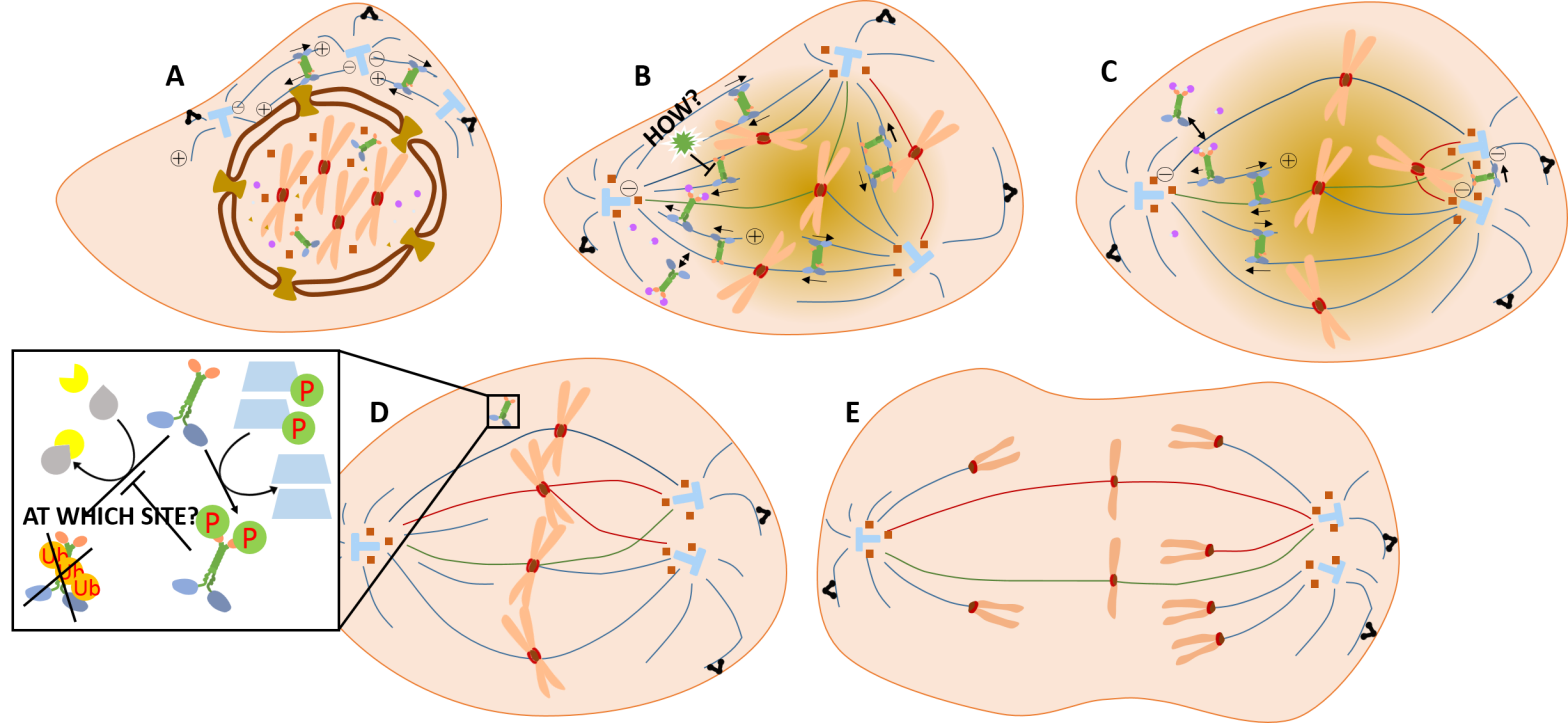
How KIFC1 fulfills its centrosome clustering activity in the cell cycle of the cancer cell **A.** Centrosomes duplicated in the interphase are moving apart in the prophase. KIFC1 has already been transported into the nucleus. **B.** Ran-GTPase probably will spatially regulate the binding of KIFC1 with microtubules by changing its gradient [61]. From the dark color to the light color in this figure, the Ran-GTPase gradient is gradually reduced. KIFC1 not only assists spindle pole organization around the centrosomes, but also clusters extra centrosomes through crosslinking and gliding [52]. **C.** During this process, NuMA helps the minus ends around the centrosomes, and dynein embeds the astral microtubule ends on the cell cortex [69, 70]. Eg5 generates an opposing force against KIFC1 in order to finely regulate the shape of the spindle [69]. **D.** In the late metaphase, KIFC1 may be degraded by APC/C. Unusual kinetochore attachments, including merotelic kinetochore microtubules and syntelic kinetochore microtubules, as regulated by SAC complexes [16, 23], further enhance bipolar spindle formation in multi-centrosome cancer cells while **E.** the rate of lagging chromosomes is increased [49].

**Figure 3:**
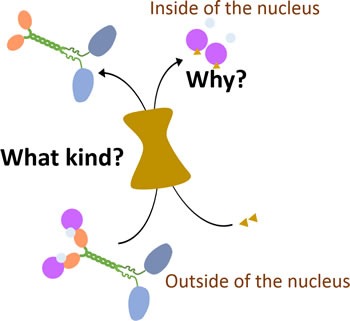
How KIFC1 is transported into the nucleus during interphase In the interphase, KIFC1 is transported into the nucleus in interphase by importin alpha/beta and Ran-GTPase through interactions with undefined nucleoporins complexes [18]. The linkages between this transportation of KIFC1 and its function in cancer cells are still missing.

**Figure 4:**
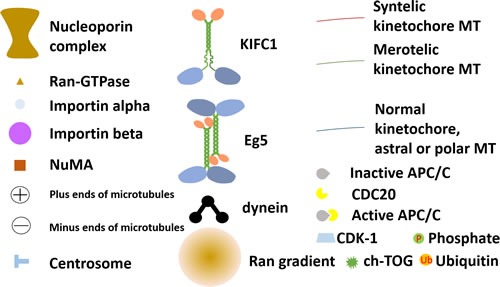
Annotations of symbols used in Figure 2 and Figure 3

So, how does KIFC1 involve in centrosome clustering processes in multi-centrosome cancer cells and how does centrosome clustering links to genetic instability of those cancer cells? A model was proposed after careful analysis of current investigations. Firstly, centrosome amplification during interphase forms a transient multipolar spindle in the prometaphase [49] (Figure 2A, 2B; See annotations of symbols of Figure 2 and Figure 3 in Figure 4). This transient multipolar spindle is thought to be the cause of the chromosome instability in cancer cells. Secondly, a merotelic kinetochore microtubule forms and acts to alter the shape of the spindle geometrically and begin to generate centrosome clustering [49]. Shortly afterwards, syntelic attachments with kinetochores appear to further promote the formation of the pseudo-bipolar spindles and complete centrosome clustering [49] (Figure 2B, 2C, 2D). Rates of the occurrence of lagging chromosomes then increase along with those of unresolved attachments, including both the merotelic attachments and syntelic ones, with kinetochores at the anaphase, leading to higher levels of chromosome instability [49, 50] (Figure 2E). During this process we deduce that KIFC1, which is able to grab the plus ends of the pre-existing microtubules [51], is transported into the nucleus, crosslinks and then slides along the antiparallel microtubules while it moves towards the spindle pole. Since diminishing of k-fiber function doesn't affect the function of KIFC1, it is assumed that KIFC1 shortens the length of the spindle independent of the k-fiber [52]. Therefore, the crosslinking between KIFC1 and microtubules happens only on polar microtubules. In this way it generates microtubule locking forces, and the locking force causes these two centrosomes to cluster together. An anaphase delay caused by increased expression of some cyclins seems to give KIFC1 the time required to transform the transient multipolar spindle into the normal bipolar spindle. In the meantime, it is KIFC1 which also triggers those changes. Research shows that knock down of KIFC1 leads to a delay of cyclin A [53]. Conversely, overexpression of KIFC1 also raises the expressions of cyclin B1, cyclin D and cyclin A which are increasing the ratio of Mad1 to Mad2, and enhancing the activity of Aurora-B kinase and shortening the whole cell cycle [23] whilst prolonging the anaphase. Moreover, this delay of anaphase onset caused by increased expression of various kinds of cyclins, as well as the presence of SAC (spindle assembly checkpoint) components such as Mad1, Mad2, BubR1, and CENP-Meta, is thought to be indispensable during the formation of unusual kinetochore microtubule attachments in humans [16, 23]. At the point of mitotic exit, when it fulfills all its responsibility, KIFC1 is found to be degraded by ubiquitination. In the human cancer cell line U2OS, KIFC1 has been found to possess an ubiquitination site which is then degraded by the 26s proteasome. Here, KIFC1 was found to be able to bind to E3 ligase APC/C at its destruction box (D box), which is also thought necessary for the degradation process [54]. In addition, CDK-1 (MPF) functions as a KIFC1 stabilizer during ubiquitination and degradation by phosphorylating KIFC1 at the Ser6 site [55] (Figure 2D). This process is somewhat converse to that of the inhibition of CDK-1 by Purvalanol A [56] which leads to the destabilization of KIFC1.

Contradictorily, even though logically it seems that KIFC1 doesn't have to be imported in to the nucleus during the interphase, there was evidence that describingnucleoporin complexes like NUP62 have shown co-expression with KIFC1 in mammalian cells including humans and mice, and in mollusks [19, 57, 58], indicating that those nucleoporin complexes might be candidates for KIFC1 to enter into the nucleus. NLS (nuclear localization signal) in the tail domain of KIFC1 is capable of directing KIFC1 to intracellular membranes [52]. In addition, KIFC1 [59] and XCTK2 [60] is known for being a vital target of Ran. More specifically, the entrance of one of the homologs of KIFC1, XCTK2, into the nucleus has been verified to be controlled by the Ran gradient in *Xenopus* egg extracts [61]. HsKIFC1 and XCTK2 were both observed to bind to alpha/beta importin [18] as well (Figure 3). Those importins can then be recycled after release [62] when transported into the nucleus during the interphase [52, 59]. HsKIFC1 is also observed to function as a replacement of XCTK2 when added into *Xenopus* egg extracts [52]. The Ran-GTP gradient is testified to be significant when XCTK2 stimulates spindle assembly by mediating the XCTK2 microtubule turnover after the release of importin from the tail of XCTK2 [61]. The understanding of the transportation regulation process relating to XCTK2 has been widely studied while that relating to HsKIFC1 still remains unclear. Although those studies on the transportation of XCTK2 can be precursors for similar research on HsKIFC1, further studies of the regulation process of translocation of HsKIFC1 are still required. Recently, depletion of a highly conserved gene, ch-TOG expression, has been seen to increase the quantity of KIFC1 which is localizing at the acentrosome spindle pole in HeLa cells [63]. This suggests that ch-TOG can limit the binding between microtubules and KIFC1, so as to decrease the functional activity of KIFC1 as a regulator in human cancer cells (Figure 2B).

Besides centrosome clustering activity and cell cycle alteration ability, by protecting cancer cell surviving signals, KIFC1 further enhances tumor malignancy (Figure 1). KIFC1 is also capable of protecting survivin from degradation by E3 ligase APC/C. KIFC1 can bind to survivin and prevents survivin from poly-ubiquitination [23], and, therefore, protects the cells from apoptosis. As mentioned before, KIFC1 itself also possess the potential to be bound and degraded by APC/C [54]. It is, therefore, possible that the protection relationship between KIFC1 and survivin is mutual. The above initiative participation roles of KIFC1 show that KIFC1 plays a significant role in actively driving tumor malignancy, and not just being elevated passively.

There are other motor proteins or proteins related to motors involved in the centrosome clustering mechanism of KIFC1. It has been suggested that a combination of the “search and capture” hypothesis [64] and the “chromatin-drive mitotic spindle formation hypothesis” [65, 66] seems to be the most likely [65], and that the kinetochore microtubule ends that originally form chromosomes can be captured by astral microtubules from the centrosomes under the transportation and microtubule locking forces of dynein [67] and the c-terminal kinesins [68]. In mammalian cells, during this kinetochore microtubule end clustering process, KIFC1 generates an opposite force against the kinesin-5 family protein Eg5, and crosslinks microtubules around the pole [69] (Figure 2B, 2C). At the same time, KIFC1's cooperation with NuMA also becomes apparent where two distinct KIFC1/NuMA- dependent processes, those of chromosome movement patterns and of the anchorage of microtubules at the spindle poles, occurs [70]. More specifically, in the first case, NuMA might gather together to form two fixed poles while KIFC1 moves along the microtubule bundle towards the minus ends, where NuMA is located, and drags chromosomes apart together with another unknown matrix element acting to anchor the other side of KIFC1. In the second case, KIFC1 along with NuMA is proposed to crosslink kinetochore microtubules and polar microtubules to convey a pole-ward force to chromosomes and thereby cause an elongation of the spindle [70]. In cancer cells, a shortening of the spindle is observed after KIFC1 knockdown [52]. This makes the second hypothesis more likely for cancer cell systems. Recently, KIFC1 along with Eg5 are also observed to act together in the transportation of γ-tubulin ring complexes (TuRCs) while it functions as a NEDD1 (GCP-WD) and augmin distribution determinant. In this way it functions to organize the minus ends [71]. Perturbation of KIF2b, a member of kinesin-13, in KIFC1/NuMA-deficient cells resulted in a normal bipolar spindle. This suggests the action of an opposite force to that which KIF2b exerts against KIFC1/NuMA [72]. The kinetochore force from the kinetochore component Nuf2 also acts as an opposing force against the centrosome clustering force of KIFC1/NuMA [73].

### Drug resistance

In cancer therapeutics, drug resistance against tubulin targeted drugs like docetaxel, taxane and tamoxifen has become an increasing problem. Studies on breast [74] and prostate cancer [75] list KIFC1 among several other factors relating to resistance.

Docetaxel, along with paclitaxel, can bind to the beta subunit of tubulin to prevent the dissociation of microtubules and to disrupt their proper organization and elongation [76]. In docetaxel resistant breast cancer cell lines, KIFC1, along with three other kinesin member proteins, KIFC3, KIF1A, and KIF5A [77], were found to be overexpressed as compared to the control group. Overexpression of KIFC3 also increases the amount of free tubulins in cells and is therefore thought to function against docetaxel by acting to depolymerize the microtubules [77]. Drugs which inhibit both KIFC1 and MCKA have been seen to act to increase prostate cancer cells' sensitivity to taxane [75].

Additional results from pre-treatment samples from basal-like breast cancer (BLBC) patients strongly suggest that KIF5A is a crucial motor protein promoting drug resistance against taxane. Its ATP-binding domain seems to be a significant factor in this process. Controversially, it has been suggested in one paper that KIFC1 might actually promote the function of taxane [78]. However, the statistical validity of this result may be called into question.

Studies in tamoxifan resistance in breast cancer cells also suggest that the abundancy of KIFC1 seems to enhance the progression of drug resistance [29].

### Redundancy in somatic cells

Although KIFC1 is found to be expressed in several tissue types and its presence in numerous bioprocess indicates its importance to both somatic and germ cells, its function is actually fairly elusive. KIFC1 is actually dispensable in ordinary somatic cells which have only one pair of non-supernumerary centrosomes. In this KIFC1 and NuMA are thought to be redundant in microtubule minus end organization [6]. It is also reported that the viability of non-multiple centrosome MCF-7 cell lines is not significantly influenced by the depletion of KIFC1 [16]. Moreover, KIFC1 has been also proven to be redundant in normal RPE1 cells (hTERT-immortalized human Retinal Pigment Epithelial cells) [47].

## POTENTIAL RISKS OF TARGETING KIFC1-RELATED SYSTEMS

### Vesicular and organelle trafficking

Electron microscopic analysis of kinesin-14 family proteins in murine dendrites confirmed the trafficking activity of the C-terminal kinesin protein KIFC2 in endocytosis [79]. What's more, the co-function and co-immunolocalization of KIF5B and KIFC1, was seen to be required for movement and fission of early endocytic vesicles. A similar function was demonstrated in 293t cell lines, with the illustration that these functions were fulfilled by opposing force between these two kinesins [17]. KIFC1 was also detected to be important for maintaining functional acidocalcisomes in *Trypanosoma brucei* through its association with acidic vesicles along the microtubules [80]. Recently, KIFC1 was found to be co-localized along with KIFC2 and dynein during endocytic vesicle transportation in human liver cells, and that Rab1a enhances KIFC1's recruitment [81] by changing Rab1a gradient [82].

### Spermiogenesis

Spermiogenesis is one of the most important mammalian bioprocess that requires KIFC1 involvement [83]. Initially, KIFC1 was found to be expressed in various tissues including the testis, heart, muscle, hepatopancreas and gill tissues of various invertebrates [84–86]. Secondly, unique KIFC1 expression patterns, that KIFC1 gather at the pole of the lone-ellipse-shaped nucleus of immature spermatids, was noted to be omnipresent and of pivotal function during specific phases of spermiogenesis in marine species [57, 84–89]. KIFC1 seems to play vital roles in two main aspects of spermatogenesis, acrosome biogenesis [18, 84, 85, 90] and nucleus deformation [84, 88, 91]. These occur *via* KIFC1's role in vesicular trafficking and minus-end transportation activities along the microtubule. KIFC1 is found to possess a vesicle association tail domain [92]. Therefore, it shouldn't be surprising to learn that KIFC1 has Golgi apparatus transportation activity during acrosome biogenesis [18]. Mechanisms of how KIFC1 generates acrosome formation is clearly illustrated in invertebrates, but how KIFC1 performs during mammalian acrosome formation needs further illustration. A special 19-amino-acid oligopeptide in the stalk domain of KIFC1, which connects with the manchette, is predicted to orientate the KIFC1 motor to vesicles like the Golgi apparatus, and this very oligopeptide is also associated with the scaffolding protein TLRR (testis leucine-rich repeat protein) [93]. TLRR was also found to possess a sequence that might bind to protein phosphatase-1 which is known to mediate the essential phosphatase modulating reversible phosphorylation of key regulators of divergent intracellular bioprocesses [94]. Those connections indicate the probability that KIFC1, with the assistance of TLRR and possibly other scaffolding proteins, is active in building a platform for the proper positioning of the regulatory factors on the nuclear membrane so as to mediate the deformation of the round spermatid nucleus and even facilitate acrosomal biogenesis.

### Oocyte development and embryo gestation

Analysis of the mechanism of somatic cell nuclear transfer (SCNT) failure in rhesus monkeys shows deficiencies in KIFC1 protein as well as Eg5 and the nuclear mitotic apparatus (NuMA) proteins [95], a matrix protein also having involvement in spindle pole assembly [69]. Such deficiencies have probably resulted from the removal of the original meiotic spindle in the oocytes. The resulting failure in SCNT led to the formation of abnormal aneuploidy embryos despite a normal spindle formation remaining around the somatic cell chromosomes [95]. Further research on SCNT using aged human oocytes showed similar failures, weak cleavages and an ineffectual formation of normal bipolar spindles around the extrinsic chromosomes in the oocytes [20]. RT-PCR studies using SCNT oocytes also revealed a poor expression of Eg5 transcripts and no sign of KIFC1, with a contrasting normal expression of KIFC1 and Eg5 in normal oocytes [20]. This acts to demonstrate the indispensable roles of such kinesins and kinesin related proteins and particularly suggests the possible pivotal role of KIFC1 for oocyte division and proper embryonic development. Moreover, KIFC1 is also reported to be divergently and periodically expressed during bovine ovarian follicular selection [96] and in the early stage human placenta, in the latter case along with KIF17 [97]. We, therefore, suspect a role of KIFC1 in gestation. Moreover, NCD was described to be essential for meiosis I [98] and II [99] in the oocytes, probably through an inhibition of the elongation of the spindle. Acentrosomal spindle formation in mouse oocytes has also been noted to involve KIFC1 [100]. It is possible that KIFC1 may have a similar function in human oocytes.

In summary, it seems that KIFC1 displays a number of fairly central functions during oocyte development and embryonic gestation. This would raise concerns for the utilization of any KIFC1 inhibitors to patients during pregnancy.

### Other possible functions of KIFC1

The active transport and binding of double-stranded DNA by KIFC1 in HeLa cells [21], as well as by NCD in an *in vitro* minimal system [101], has also been reported. Transporting exogenous DNA into the nucleus has been long used as a strategy of gene therapy. However, whether the motion of this transportation was of thermodynamic diffusion or of motor trafficking remained unknown until a study demonstrated that the depletion of KIFC1 dramatically decreased the motility of DNA in HeLa cells *in vitro* [21]. Conversely, inhibition of three plus-end kinesins, also co-purified with DNA in cell extracts using SDS-screening methods, had no significant impact on the transportation of double-stranded DNA [21]. This indicates that the exogenous double-strand DNA transportation activity of KIFC1 may not be replaceable.

Despite the centrosome clustering function in cancer cells, KIFC1 is also observed to have a similar function in human primary lung fibroblast cell lines [53]. Another case showed that injecting antibodies against KIFC1 along with those against NuMA disturbed a proper formation of the bipolar spindle. However, this disturbance of spindle shape could be rescued by perturbation of static force conducted by the kinetochore component Nuf2 in KIFC1/NuMA-deficient cells [73]. Nevertheless, we dismissed the conclusion drawn from the second case for several reasons. Firstly, they failed to show what happens if KIFC1 alone is knocked down in somatic cells. Therefore, they did not rule out the possibility that the disturbance of spindle shape was simply caused by NuMA alone. Secondly, the conclusion only proves that the motor force converted by KIFC1 and/or NuMA acts as an antagonist to that of the static force provided by certain kinetochore components.

A knockdown of KIFC1 would render the drug ineffectiveness of taxol, which is elucidated in an *in vivo* study of an African Green Monkey kidney (BSC 1) cell line where the cells were constitutively able to express GFP-tubulin [102]. This process involved the recruitment of KIFC1. Under the treatment of taxol, it is at the G2/M checkpoint of the cell cycle that microtubules are observed to be released from the centrosomes and move to the cortex. This is due to the inability of the centrosomes to hold onto successively growing microtubules. Then, microtubule cross-linking protein NuMA and KIFC1 are recruited and co-function at the ends of the microtubules that were embedded on the cell cortex. Finally, the microtubules start to move. They first form a curved sheet and then become c-shaped or move into open arrays, ultimately gathering into hollow cytasters. Those cytasters aid in multipolar divisions which eventually lead to cell death. NuMA may also assist in the release of the microtubules from the centrosomes by embedding the minus ends into the cell cortex [102]. It is noteworthy that dynein, which also possesses minus-end microtubule trafficking activity, seems not to be influential in this process.

## CHEMOTHERAPIES AGAINST KIFC1

Kinesins including KIFC1 bind to microtubules with their motor domains, and ATP hydrolysis sites [103] included by these motor domains are popular disturbing sites for designed drugs. As KIFC1 has emerged popular as a chemotherapy target, three small-molecule inhibitors of KIFC1 have been thus far highlighted. There are two direct inhibitors of KIFC1, AZ82 and CW069. CW069 has already shown high specificity to KIFC1, making it a desirable clinical drug candidate. PJ34, which is likely to suppress KIFC1 transcriptionally, may also be a promising option.

### AZ82

AZ82 binds to the KIFC1-microtubule complex and inhibits the ATP-hydrolysis activity of KIFC1. More specifically, AZ82 blocks the release of ADP and the reception of ATP, thus cutting off the power supply for KIFC1's gliding along the microtubule [104]. When cancer cells with extra centrosomes are treated with AZ82, fatal multipole spindles appears. However, an overdose of AZ82 can lower the selective efficiency of such a drug [104]. Moreover, intraperitoneal injections of AZ82 in mice demonstrated reasonable half-lives. It is this long drug sustainability which is an essential quality for qualification as a prescription drug [105]. Nonetheless, AZ82 also rescues phenotypes induced by deficiency of kinesin-5 Eg5 [104, 105], which is understandable as the KIFC1 functionally provides an opposing force against that offered by Eg5. This provides a warning to avoid any combination of KIFC1 inhibitors and Eg5 inhibitors.

### CW069

CW069 is a highly selective small-molecule KIFC1 inhibitor with an affinity to the loop 5 cleft of its globular motor domain which disrupts KIFC1's ability to drive the motility of microtubules [106]. *In vivo* test results of CW069 proved lethal to breast cancer cell lines while the spindle shape of the matched group, normal dermal fibroblast cells, were not significantly altered. In addition, although there is an up to 80% protein similarity between the motor domains of KIFC1 and KSP (kinesin spindle protein), the specificity of CW069 is sufficient to avoid any mitotic phenotype that would occur upon the inhibition of KSP [106]. This makes its action more predictable and it is likely to end up as a licensed drug. Still, other investigations on CW069 have further verified its potential to be a reliable chemotherapy tool [107].

### PJ34

Upon the discovery of PJ34 (phenanthridines), it quickly became known for its poly-(ADPribose) polymerase (PARP) inhibition activity, despite its target profile being not quite complete [108]. Recently, PJ34 turns out to be a cancer cell terminator which does little harm to normal cells. It has been specifically reported to possess a centrosome de-clustering function [109]. The mechanisms of how PJ34 intervene in cases of multiple centrosome clustering remain unknown. Likewise, co-immunoprecipitation results of PJ34 and KIFC1 have yet to be reported. Due to the fact that the KIFC1 mRNA level is shown to be significantly diminished in various PJ34 treated breast cancer cell lines [110], we speculate that PJ34 might be transcriptionally suppressing the expression of KIFC1 given that PARP has been formerly reported to be a transcriptional regulator [111].

## CONCLUSIONS AND PERSPECTIVES

Research both for and against KIFC1 as a chemotherapy target has been discussed and the functions of the kinesin-14 proteins mentioned in this article are summarized in Table 1. What should be emphasized is that research on the endocytic vesicle trafficking role of KIFC1, discussed as a potential barrier towards the use of KIFC1 inhibitors, has included evidence from cancer cell lines. Since we don't know whether endocytic vesicle trafficking is relevant to unique metabolism processes of cancer cells and whether knockdown of KIFC1 in those cell lines causes higher death rates compared to that of knockdown of proper control cells, those experiments could actually be considered as evidence for either side based on different arguments. Similarly, we must consider that research showing that KIFC1 increases cyclin A, as is discussed as a clear advantage for the potential use of a KIFC1 inhibitors against cancer, was partly concluded in fibroblast cells.

**Table 1:** Kinesin-14 functions in animals:

Protein	Cell type	Species	Function	Status	Reference
KIFC1	293t cell line	Human	Moving and assist fission of early endocytic vesicles	--	[17]
	Somatic cell	Trypanosoma brucei	Maintaining functional acidocalcisomes	Con	[80]
	Hepatoma cell line	Human	Moving endocytic vesicles	--	[81]
	Testis	Various marine organisms	Trafficking Golgi apparatus during acrosome formation	Con	[18, 93]
	Oocytes	Rhesus	Organizing meiotic spindles	Con	[95]
	Oocytes	Human	Organizing bipolar spindle and promote cell cleavage	Con	[69]
	Oocytes	Mouse	Organizing acentrosomal spindle formation	Con	[100]
	Ovary	Bovine	Ovarian follicular selection	Con	[96]
	Placenta	Human	Gestation	Con	[97]
	HeLa cells	Human	Double-strand DNA transportation into nucleus	Con	[101]
	primary lung fibroblast cell line	Human	Acentrosomal spindle organization	Con	[53]
	non-small-cell lung cancer (NSCLC)	Human	Altering cytoskeleton and promoting tumor metastasis	Pro	[25]
	Ovarian cancer	Human	Altering cytoskeleton and promoting tumor metastasis	Pro	[22]
	Multi-centrosome cancer cells	Human	Centrosome clustering in multi-centrosome	Pro	[16, 22, 23]
	Fibroblast IMR-90 cell	Human	Regulation of cyclin A	--	[53]
	Breast cancer	Human	Regulation of cyclins	Pro	[23]
	Breast cancer	Human	Protect survivin from degradation	Pro	[23]
	Breast cancer	Human	Docetaxel resistance	Pro	[77]
	Kidney cell line BSC 1	African green monkey	Improving taxol effectiveness and elevating taxol-driven cell death	Con	[102]
KIFC2	Dendrite cell	Mouse	Trafficking multi-vesicular body-like organelles	Con	[79]
	Hepatoma cell line	Human	Moving endocytic vesicles	Con	[81]
KIFC3	Breast cancer	Human	Docetaxel resistance	Pro	[77]
NCD	Oocytes	Drosophila	Spindle shaping of meiosis I and II		[98, 99]

After careful comparison we suggest that KIFC1 can be a promising chemotherapy target. On the one hand, side effects of KIFC1 inhibitors are theoretically avoidable. Functions that KIFC1 possesses in normal cells, including promoting spermiogenesis, vesicular transportation and acentrosomal spindle formation, seem to be vital. But the presence of KIFC1 is limited to certain cell types: germ cells and fibroblast cells, and KIFC1 is still redundant in most somatic cells who won't go on acentrosomal cell division. On the other hand, the indispensability and importance of KIFC1 during mitosis of cancer cells as well as its dispensability in general bioprocesses of normal somatic cells reveals its great potential of being a chemotherapy target. By ensuring a certain level of supernumerary centrosomes and genetic instability, KIFC1 also assists in the enhancement of tumor malignancy. Besides, KIFC1 possesses the ability to initiatively delay anaphase to gain sufficient time to fulfill centrosome clustering ability by increasing the expression of cyclin B1, cyclin D, cyclin A, perturbing the balance between Mad1, Mad2, and elevating the activity of Aurora-B kinase. By protecting survivin from ubiquitination, KIFC1 provides accessory assistance for tumor malignancy. These functions of KIFC1 in cancer cells demonstrate its importance and indispensability during cancer cell survival and malignancy.

While recent data shows a clear picture of the degradation process of KIFC1 by E3 APC/C and the protection effect from CDK-1 to KIFC1 by phosphorylation (Figure 2D), further investments for the regulation of expression processes is required for the sake of developing more indirect inhibitors. Current investments suggest that the E2alpha stimulation pathway and its activation of ANCCA and p110 CUX1 in estrogen-receptor positive tumors might be intriguing candidates for researches on transcription processes of the KIFC1 gene. The linkages between the importation of KIFC1 into the nucleus and its centrosome clustering and cell cycle disturbing function are still missing as it seems unnecessary for KIFC1 to resume its bio-function in cancer cells (Figure 3). Moreover, how Ran-GTPase gradient and importin alpha/beta regulate spatial distribution of KIFC1 and what kinds of nucleoporin complexes especially in human cancer cells, and how ch-TOG limits the function of KIFC1 are as yet unknown (Figure 2B).
